# Conserved *Streptococcus pneumoniae* Spirosomes Suggest a Single Type of Transformation Pilus in Competence

**DOI:** 10.1371/journal.ppat.1004835

**Published:** 2015-04-15

**Authors:** Raphaël Laurenceau, Petya V. Krasteva, Amy Diallo, Sahra Ouarti, Magalie Duchateau, Christian Malosse, Julia Chamot-Rooke, Rémi Fronzes

**Affiliations:** 1 Unité G5 Biologie Structurale de la Sécrétion Bactérienne, Institut Pasteur, Paris, France; 2 UMR 3528, CNRS, Institut Pasteur, Paris, France; 3 Spectrométrie de Masse Structurale et Protéomique, Institut Pasteur, Paris, France; 4 Plate-Forme de Protéomique, Institut Pasteur, Paris, France; The University of Texas Health Science Center at San Antonio, UNITED STATES

## Abstract

The success of *S*. *pneumoniae* as a major human pathogen is largely due to its remarkable genomic plasticity, allowing efficient escape from antimicrobials action and host immune response. Natural transformation, or the active uptake and chromosomal integration of exogenous DNA during the transitory differentiated state competence, is the main mechanism for horizontal gene transfer and genomic makeover in pneumococci. Although transforming DNA has been proposed to be captured by Type 4 pili (T4P) in Gram-negative bacteria, and a competence-inducible *comG* operon encoding proteins homologous to T4P-biogenesis components is present in transformable Gram-positive bacteria, a prevailing hypothesis has been that *S*. *pneumoniae* assembles only short pseudopili to destabilize the cell wall for DNA entry. We recently identified a micrometer-sized T4P-like pilus on competent pneumococci, which likely serves as initial DNA receptor. A subsequent study, however, visualized a different structure - short, ‘plaited’ polymers - released in the medium of competent *S*. *pneumoniae*. Biochemical observation of concurrent pilin secretion led the authors to propose that the ‘plaited’ structures correspond to transformation pili acting as peptidoglycan drills that leave DNA entry pores upon secretion. Here we show that the ‘plaited’ filaments are not related to natural transformation as they are released by non-competent pneumococci, as well as by cells with disrupted pilus biogenesis components. Combining electron microscopy visualization with structural, biochemical and proteomic analyses, we further identify the ‘plaited’ polymers as spirosomes: macromolecular assemblies of the fermentative acetaldehyde-alcohol dehydrogenase enzyme AdhE that is well conserved in a broad range of Gram-positive and Gram-negative bacteria.

## Author Summary

*Streptococcus pneumoniae* often escapes prevention and treatment through rapid horizontal gene transfer via natural transformation. Uptake of exogenous DNA requires expression of a transformation pilus but two markedly different models for pilus assembly and function have been proposed. We previously reported a long, Type 4 pilus-like appendage on the surface of competent pneumococci that binds extracellular DNA as initial receptor, while a separate study proposed that secreted short, ‘plaited’ transformation pili act simply as peptidoglycan drills to open DNA gateways. Here we show that the ‘plaited’ structures are not competence-specific or related to transformation. We further demonstrate that these are macromolecular assemblies of the metabolic enzyme acetaldehyde-alcohol dehydrogenase—or spirosomes—broadly conserved across the bacterial kingdom.

## Introduction

Despite medical advances and vaccination campaigns, respiratory tract invasion by *Streptococcus pneumoniae* remains a leading mortality cause worldwide [1–3]. A particular challenge in the prevention and treatment of pneumococcal infections lies in the bacterium’s striking genomic plasticity, as it allows for efficient antibiotic resistance development, capsular serotype switching and vaccine escape [4]. Horizontal gene transfer and chromosomal rearrangements typically result from the avid uptake and recombination of exogenous DNA known as natural transformation. A strictly regulated event, it occurs during a transitory state of the bacterium’s life cycle—competence—and requires the timed expression of a dedicated set of genes [5]. Among these are the genes of the *comG* operon, which are conserved among naturally competent Gram-positive bacteria and are homologous to the ones encoding Type 4 pili (T4P) and Type 2 secretion system (T2SS) pseudo-pili components in Gram-negative bacteria [6,7]. Although mechanistic studies of structural determinants for DNA uptake—such as putative transformation-specific cellular appendages—hold promise for the development of novel antiinfectives and helper compounds, there have been only limited and contradictory reports on the initial steps of this important biological process [8–10].

As until recently no pilus-like structure had been observed in any transformable Gram-positive bacterium, it had been postulated that the pneumococcal *comG* operon encodes a short T2SS-like pseudo-pilus that serves to destabilize the cell wall peptidoglycan for DNA entry [6,9,11]. The main experimental evidence for this model comes from a different transformable organism, *Bacillus subtilis*, where pilus length was indirectly deduced from biochemical data [9].

**Fig 1:**
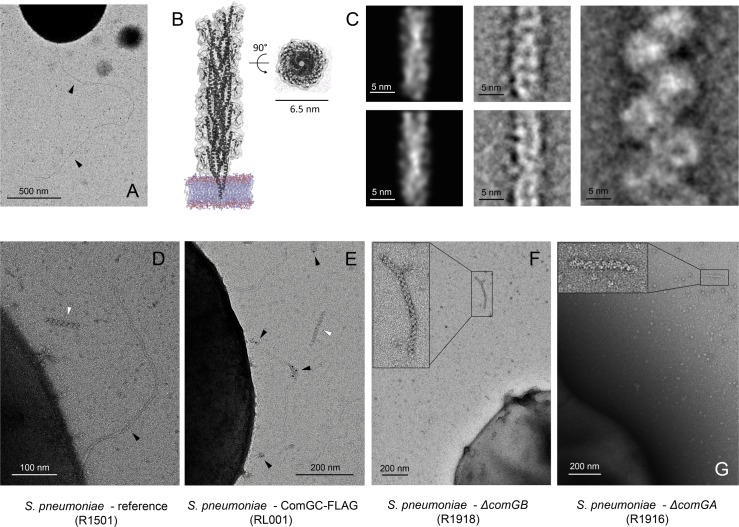
Pneumococcal transformation pilus controversy: Long, T4P-like transformation pilus versus short, ‘plaited’ polymers. **(A)** Long, T4P-like transformation pilus protruding on the surface of competence-induced *S*. *pneumoniae* strain R1501 [10]. **(B)** High-resolution structural model of a T2SS-peudopilus in side (left) and top (right) views [15], visualized in PyMOL (Schrödinger). **(C)** Scaled electron density reprojections of the T2SS-pseudopilus model (left), compared to class averages of the long T4P-like transformation pilus reported in [10] (center) and the short, ‘plaited’ filaments reported in [8] (right). Scale bars 5 nm. **(D)** Coexistence of the T4P-like pilus (black arrowhead) and ‘plaited’ filaments (white arrowhead) in competence-induced *S*. *pneumoniae* culture. **(E)** Immunogold labeling of major pilin ComGC in a strain carrying an additional FLAG-tagged ectopic copy of the *comGC* gene. A T4P-like pilus and a ‘plaited’ filament are indicated by black and white arrowheads, respectively. **(F)** and **(G)** Unabolished release of ‘plaited’ filaments in non-competent, pilus-deficient *ΔcomGB* [17] and *ΔcomGA S*. *pneumoniae*, respectively.

Our team identified a long, micrometer-sized, T4P-like pilus protruding on the surface of competent cells from different pneumococcal strains with wild-type genotype (Fig 1*A*) [10]. Among these are two highly transformable laboratory strains of different genetic background—R6 and TCP1251—as well as a capsulated clinical isolate—the G54 strain [10]. We showed that major constituent of the transformation pilus is the ComGC pilin and that the pilus is sensitive to mechanical stress, which can lead to its detachment from the cell (shearing) [10,12]. Finally, we showed that this transformation pilus binds extracellular DNA and proposed that it acts as the initial DNA receptor on the surface of competent pneumococci [10].

A subsequent study visualized completely different structures—short, ‘plaited’ polymers—in the medium of competence-induced *S*. *pneumoniae* [8]. Biochemical observation of significant ComGC release in the medium during competence convinced the authors that the ‘plaited’ structures corresponded to secreted transformation pili. After failing to immunolabel these structures, they expressed heterologously the whole *comG* operon in *Escherichia coli* and visualized the release of similar polymers [8]. Finally, they proposed a model, which is consistent with the classical but speculative model of transformation pseudo-pili: rather than acting as a DNA receptor, the pneumococcal transformation pilus acts as a peptidoglycan-drilling device whose release leaves a gateway for transforming DNA to find the uptake machinery [8,10].

Here we show definitive experimental evidence that the short ‘plaited’ filaments are not transformation pili or other structural determinants of natural transformation. We further identify the structures as fermentative spirosomes, or macromolecular complexes of the acetaldehyde-alcohol dehydrogenase enzyme AdhE, which is widely conserved across the bacterial kingdom. Being aware of the limited view and resolution that observation by electron microscopy provides, we underscore the need for thorough validation by orthogonal approaches. Finally, we briefly synthesize the present-day published collective knowledge by proposing an updated model of pneumococcal transformation.

## Results

### T4P-like pilus vs ‘plaited’ filaments: Morphological differences

Perhaps the most intriguing aspect of the Balaban *et al*. study is the distinctive morphology of the reported ‘plaited’ filaments themselves [8]. As the authors point out, the genetic makeup of the *comG* operon resembles significantly that of operons encoding T4P or T2SS components in Gram-negative bacteria [5,7,8,13]. This includes from sequence homology of the individual genes through their intraoperon organization to the putative bioassembly platform and post-translational modifications of the encoded components. The structure of both T4 pili and T2SS pseudo-pili has been extensively studied [14,15]. Generally T4 pilins pack tightly into thin but extremely strong and several micrometers long surface-attached helical filaments [14]. Typical dimensions vary from 5–6 nm width for the T4aP of many bacteria (*Pseudomonas aeurginosa*, *Neisseria gonorrhoeae* and others) to the thicker, about 8 nm wide T4bP of enteropathogens such as *Vibrio cholerae* and *Salmonella enterica* serovar Typhi [14]. Conversely, structural models of T2SS pseudopili, which normally act as short, protein ejecting pistons in the periplasm, present an architecture that is very similar to that of gonococcal T4aP (Fig 1*B*) [15]. Both T4P and T2SS pseudo-pili feature a grooved surface with relatively small protuberations characteristic of the pilin helical packing [14,15].

The characteristic structural features of T4P were conserved in the transformation pili that we visualized previously on the surface of competence-induced pneumococci from several different wild-type strains [10]. In contrast, the ‘plaited’ structures visualized subsequently represent short, 40–200 nm long structures that are significantly thicker (~ 10 nm) and present large protuberant domains on their helical surface (Fig 1*C*) [8]. The authors proposed that the filaments are ‘plaited’, i.e. composed of two interlaced transformation pili [8]. Given the tight pilin packing in known T4P structures, however, we found it quite striking that homologous pili could sustain such a significant deformation to form an interlaced dimer. Although molecular dynamics simulations revealed that T2SS pseudo-pili can adopt a wide range of helical twist angles, they were not shown to have a propensity for short-range bending [16]. Moreover, while many T4P can form bundles, those can be hundreds of nanometers thick, contain a large number of pili and present much more limited distortion at the level of individual filaments [14].

### T4P-like and plaited filaments co-exist in competent *S*. *pneumoniae* cultures

Intrigued by the striking new features of the ‘plaited’ filaments we wanted to see whether the transformation pili we previously reported could form similar structures. We were able to visualize the ‘plaited’ polymers along with the T4P-like long transformation pili in wild-type pneumococci (Fig 1*D*). However, in a strain with an additional FLAG-tagged ectopic copy of *comGC* gene for the major pilin [10] (S1 Table), we were able to immunolabel only the long, surface-attached pili using an anti-FLAG antibody (Fig 1*E*). Similar results were reported by Balaban and colleagues who failed to immunolabel the ‘plaited’ structures with a different antibody raised against ComGC [8].

### The ‘plaited’ filaments observed in *S*. *pneumoniae* cultures are not related to pilus biogenesis or competence

**Fig 2:**
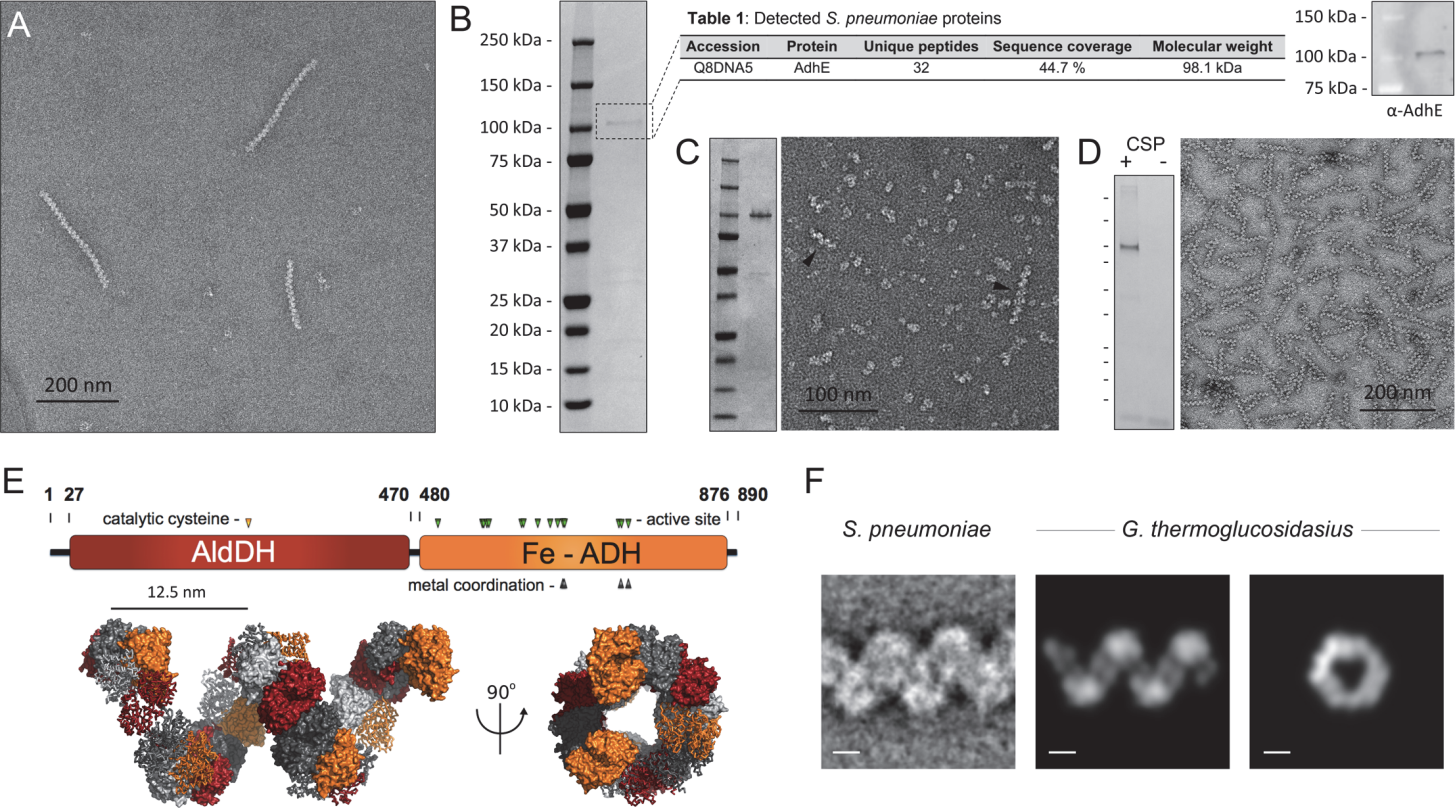
Identification of the ‘plaited’ filaments as AdhE spirosomes. **(A)** Negatively stained purified ‘plaited’ filaments. **(B)** Left, coomassie stained SDS-PAGE on the purified filament fraction. Center, proteomic analysis of the predominant protein band; Posterior Error Probability (PEP) value for AdhE is 0. Right, Western blot validation using an anti-AdhE antibody (Agrisera). **(C)** Heterologous expression in *E*. *coli* and purification of AdhE^S.pneumoniae^. Left, coomassie stained SDS-PAGE of the affinity purified protein (S2 Table). Right, negatively stained electron microscopy of the purified protein with black arrowheads indicating spontaneously formed spirosomes. **(D)** Immunopurification of *S*. *pneumoniae* spirosomes from a strain carrying an additional competence-inducible FLAG-tagged ectopic copy of the *adhE* gene (SO007): left, coomassie-stained SDS-PAGE of the eluted fraction in CSP-induced and-uninduced cells (CSP, competence stimulating peptide); right, negative-stain EM on the eluted fraction **(E)** Top, AdhE domain organization; bottom, high-resolution structural model of a *G*. *thermoglucosidasius* spirosome [19], visualized in PyMOL (Schrödinger). The color of the individual protomers alternate between grayscale and domain-coded color representation; successive AdhE dimers alternate between ribbon and surface representation **(F)** A representative class average of the ‘plaited’ filaments compared to electron density reprojections of the *G*. *thermoglucosidasius* spirosome model [19]. Scale bars 5nm.

As negative immunolabeling results are difficult to interpret and can be due to a variety of technical and structural factors, we proceeded to investigate the role of the plaited filaments in competence. We tested three negative control strains carrying either single-gene deletions for essential pilus biogenesis components (S1 Table)—the assembly platform protein ComGB [17] or the associated powering ATP-ase ComGA—or expressing a preprocessing incompetent ComGC variant (ComGC^E20V^, or ComGC^E5V^ in mature pilin residue numbering). As shown previously, although these mutants can express monomeric pilins, they cannot assemble surface exposed pili and are transformation-incompetent [8,10,17]. In all three *ΔcomGA*, *ΔcomGB* and *comGC* ^*E20V*^ strains we still detected release of ‘plaited’ filaments while expression of the long, T4P-like transformation pilus was abolished (Fig 1*F* and Fig 1*G* and S1). Finally, we observed significant release of these structures even in the absence of competence induction (Fig 2A), further confirming that they are not related to pilus biogenesis during natural transformation.

### The ‘plaited’ filaments observed in *S*. *pneumoniae* cultures are spirosomes—Macromolecular complexes of the fermentative enzyme AdhE

To identify the building subunits of the ‘plaited’ filaments, we developed an enrichment and purification protocol based on differential ultracentrifugation, microfiltration and size exclusion chromatography. Electron microscopy imaging of the purified polymers showed a homogeneous sample composed primarily of the characteristic coiled polymers with an average length of 100–300 nm (Fig 2*A*). SDS-PAGE analysis of the corresponding fraction showed the presence of a predominant protein species with a molecular weight of ~100 kDa. LC-MS/MS proteomic analyses on the excised, trypsin-digested gel band, as well as on the total purified filaments fraction (S2 Table), unambiguously identified the predominant protein as bifunctional acetaldehyde-alcohol dehydrogenase AdhE, and the result was validated biochemically by Western blot detection using an anti-AdhE antibody (Fig 2*B*). Heterologous expression of the *S*. *pneumoniae adhE* gene in *Escherichia coli* and affinity purification of the expressed protein showed spontaneous coiled filament formation in the eluted fraction (Fig 2*C*). Finally, the protein composition of the ‘plaited’ polymers was validated by affinity pull-down with anti-FLAG antibody-conjugated resin on samples from a *S*. *pneumoniae* strain carrying an additional ectopic *adhE* gene copy for competence-inducible expression of a C-terminally FLAG-tagged protein (Fig 2*D*).

AdhE is a 98 kDa protein with an N-terminal acetylating aldehyde dehydrogenase domain (AldDH) and a C-terminal Fe-dependent alcohol dehydrogenase domain (Fe-ADH) (Fig 2*E*) [18]. Homologous dual domain proteins are common among fermentative bacteria and are reported to catalyze the NADH-dependent conversion of acetyl-CoA to ethanol via an aldehyde intermediate. Most importantly, in many species AdhE has been shown to polymerize into fine helical filaments called spirosomes [19–26] that are morphologically consistent with the ‘plaited’ filaments discussed here and reported as self-secreting, ‘plaited’ transformation pili by Balaban and colleagues [8]. A high resolution structural model of spirosome assembly by the closely homologous AdhE of *Geobacillus thermoglucosidasius* shows multimeric arrangement of the individual subunits into a right-handed spiral filament with six protomers per helical turn and overall pitch and width parameters consistent with negatively stained class averages of its pneumococcal counterpart (Fig 2*E* and 2*F*) [19]. It is also important to note that the proposed spirosome structure—which is based on crystallographic and in-solution biophysical data, homology modeling and *in silico* macromolecular docking—corresponds to a single-start helix rather than a ‘plaited’ polymer of two or more interlaced filaments [19].

### AdhE is not required for natural transformation

**Fig 3:**
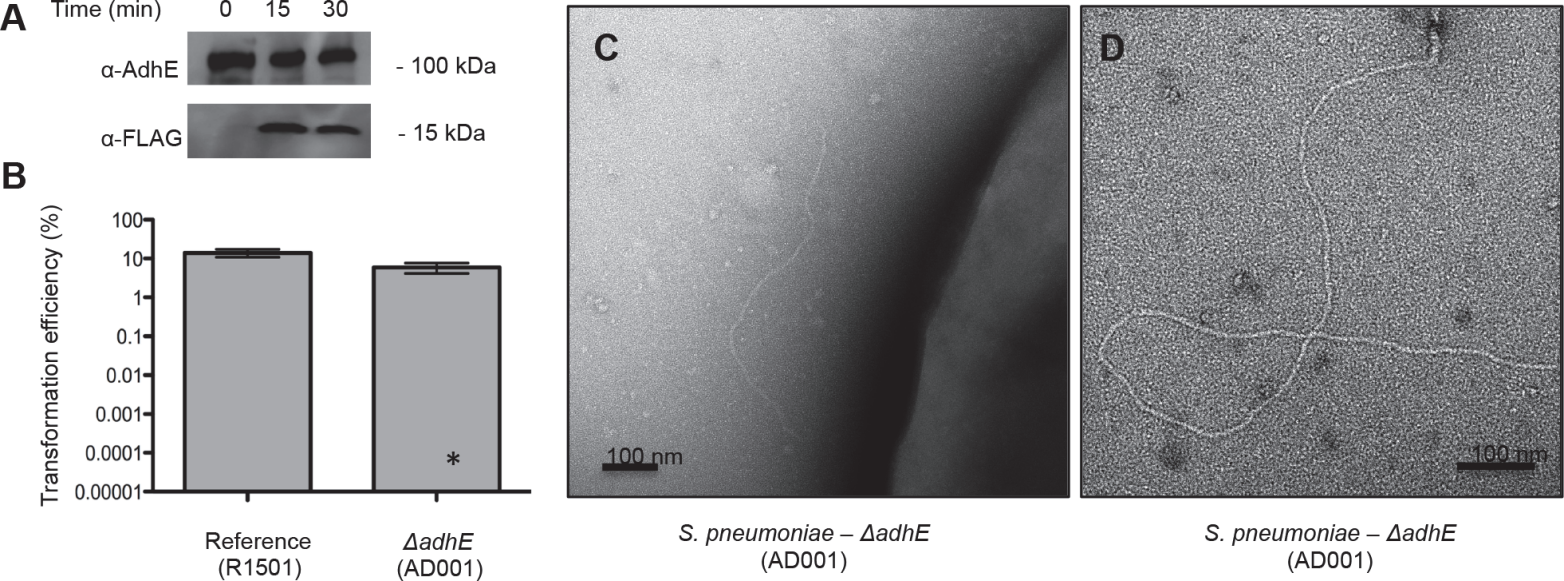
AdhE during competence induction. (**A**) Expression levels of AdhE and ComGC-FLAG in the RL001 strain following competence induction using an anti-AdhE and anti-FLAG antibodies, respectively. Samples were normalized for total protein content prior to loading on the gel. (**B**) Transformation efficiency of an *adhE*-null (strain AD001) mutant relative to the reference strain (strain R1501). The data are representative of two biological replicates, with two repetitions each. An asterisk indicates typical transformation efficiency upon ComGC disruption [10]. (**C-D**) T4P-like transformation pili assembled by the *ΔadhE* strain.

To examine a putative role of AdhE in natural transformation, we first followed the protein’s expression over the course of competence induction that was verified by the detection of a competence-inducible FLAG-tagged ectopic copy of ComGC. While we have shown competence-specific ComGC expression in wild-type genetic background previously [10], AdhE protein levels remained stable over the course of the experiment (Fig 3*A*). We next constructed an *adhE*-null *Streptococcus pneumoniae* R6 mutant (*ΔadhE*) and examined its transformation efficiency for uptake of resistance-encoding DNA cassette under challenge with the corresponding antibiotic. While the *ΔadhE* mutant shows slightly decreased transformation efficiency (~ 2-fold), this change is negligible compared to typical results under comGC disruption (~ 10 000-fold) and can be due to reduced metabolic fitness under the microaerobic conditions of the experiment (Fig 3*B*). Our data are consistent with a previous genome-wide study aiming to identify genes essential for natural transformation in *Streptococcus pneumoniae*, which have failed to identify AdhE as a requirement for DNA uptake [27]. Finally, while no spirosome release was detected for the competence-induced *ΔadhE* pneumococci, typical T4P-like transformation pili were observed (Fig 3*C* and 3*D*).

### Spirosome formation is a conserved metabolic phenomenon across the bacterial kingdom

Formation of spirosomes has been reported in a variety of Gram-positive and Gram-negative bacteria, with the first studies dating back several decades and refering to the building protein, AdhE, as spirosin [19–26]. AdhE conservation across representative species with confirmed spirosome formation shows significant sequence homology even among relatively distant taxa (Table 1). Nevertheless, sequence similarity mapping along the AdhE^*G*.*thermoglucosidasius*^ structural model reveals that highly conserved residues cluster in only few surface-exposed patches [19,28]. These correspond to the deep active site clefts of the two dehydrogenase domains, as well as sites at or near the interdomain linker. The latter would likely remain buried in the context of mature spirosomes, as they stabilize embrace-like interactions between AdhE monomers in the high-resolution structural model of *G*. *thermoglucosidasius* spirosomes [19] (S2 Fig). Thus the exposed spirosome surface would retain significant variability, which in turn could translate into differences in spirosome morphology and stability across species. Moreover, earlier reports have demonstrated that spirosome helix parameters can vary significantly depending on the presence and type of small molecule and metal ion cofactors [20,21].

**Table 1:** AdhE sequence conservation in representative Gram-positive (+) and Gram-negative (-) species with confirmed spirosome formation [19–26].

Accession	Organism	Identity	Similarity
Q8DNA5	*Streptococcus pneumoniae* (+)	100%	100%
A3CK27	*Streptococcus sanguinis* (+)	96%	98%
F8CW95	*Geobacillus thermoglucosidasius* (+)	58%	75%
Q03B11	*Lactobacillus casei* (+)	55%	72%
M5AAG2	*Lactobacillus brevis* (+)	54%	71%
P0A9Q7	*Escherichia coli* (-)	48%	66%
N1K5R4	*Yersinia enterocolitica* (-)	47%	65%
E7NR96	*Treponema phagedenis* (-)	47%	65%
Q18D77	*Clostridium difficile* (+)	47%	64%
A4ECL0	*Collinsella aerofaciens* (+)	47%	63%

**Fig 4:**
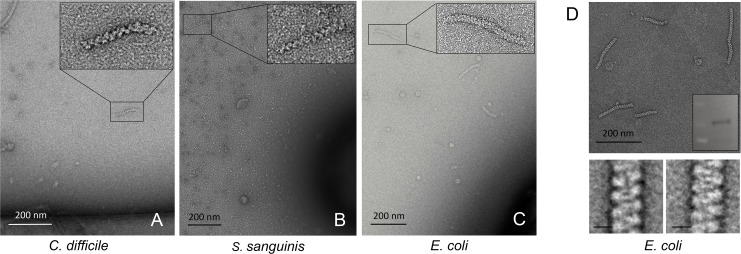
Cross-species conservation of AdhE spirosomes. **(A-C)** Negative-stain electron microscopy on spirosomes released in cultures of *C*. *difficile*, *S*. *sanguinis* and *E*. *coli* strains [29,30]. **(D)** Purification (top), AdhE immunodetection (inset) and representative class averages of *E*. *coli* spirosomes; scale bars 10 nm (bottom).

In addition to *S*. *pneumoniae*, we observed spirosome release in cultures of *Clostridium difficile*, *Streptococcus sanguinis*, and *E*. *coli* (Fig 4*A–4C* and Table 1) [29,30]. An *adhE* null strain of *S*. *sanguinis* [30] showed no release of morphologically consistent filaments, serving as an additional control for correct target identification. For the two Gram-positive species, *C*. *difficile* and *S*. *sanguinis*, spirosome morphology was practically indistinguishable from that of *S*. *pneumoniae*. The helical filaments we observed in *E*. *coli* cultures, on the other hand, were visibly more tightly coiled (Fig 4D) To verify that those correspond to AdhE macromolecular complexes we purified an enriched spirosome fraction and validated its major component as the bifunctional dehydrogenase using proteomic and biochemical methods (Fig 4D and S3 Table). Thus, although we expect morphological variations to be commonplace across species and even sample handling protocols, we are confident that spirosome assembly and extracellular release can be detected in many more environmental, clinically isolated, or genetically engineered bacterial strains.

## Discussion

### *S*. *pneumoniae* and *E*. *coli* release helical AdhE spirosomes and not plaited transformation pili

The major horizontal gene transfer mechanism in *S*. *pneumoniae*—natural transformation—requires regulated expression of the *comG* pilus biogenesis operon, homologous to operons encoding T4P and T2SS pseudopili in Gram-negative bacteria [5,7,8,13]. Recent studies have reported conflicting results regarding the morphology and function of pneumococcal transformation pili. One proposed mechanism is that *S*. *pneumoniae* expresses a long, DNA-binding, T4P-like cell surface appendage to ‘fish’ extracellular transforming DNA [10], while an alternative hypothesis argues that competent pneumococci express short, self-secreting T2SS plaited pili that perforate the cell wall peptidoglycan to allow for DNA entry [8].

Here we show that the ‘plaited’ filamentous polymers are not related to natural transformation or pilus biogenesis but are instead widely conserved and well documented macromolecular complexes of the fermentative enzyme bifunctional acetaldehyde-alcohol dehydrogenase AdhE. Its tandem domain architecture secures the two-step NADH-dependent reduction of acetyl-CoA to ethanol via an aldehyde intermediate [19–25,31]. Although the biological significance of AdhE polymerization in such massive structures remains enigmatic, it is plausible that spirosome assembly delivers spatially localized metabolic flux to limit diffusion of the highly reactive aldehyde species and secure optimized conversion kinetics [32]. In addition, the high resolution structural model of *G*. *thermoglucosidasius* spirosomes shows that polymer assembly buries ~ 6 500 Å^2^ of surface area per monomer, which could have dramatic effects on protein stability and function [19,31].

Extracellular spirosome release by cultured bacteria appears to be the result of random cell lysis as no biological function or secretion mechanism can be assigned to the phenomenon. As expected for a fermentative enzyme and consistent with reports in the literature, AdhE expression and spirosome assembly is expected to increase under microaerobic and anaerobic conditions as opposed to aerobic cultures [23,33]. Anaerobic growth and increased cell lysis are both common in cultures of competence-induced pneumococci, where the signaling process of fratricide kills non-competent cells to release extracellular DNA available for uptake [17,34]. This can explain why the spirosomes were initially associated to natural transformation in *S*. *pneumoniae* and reported to represent ‘plaited’ transformation pili [8]. Conversely, in their study Balaban and colleagues attempted to reconstitute expression of pneumococcal transformation pili in *E*. *coli* by heterologous expression of the entire *comG* operon [8]. As a result, extracellular release of spirosomes was readily detected and the AdhE polymers were again labeled erroneously. It remains unclear why the authors failed to detect spirosomes in their negative control culture. One possibility is that the structures were omitted in the limited observation field that high-magnification electron microscopy experiments provide. Conversely, it is possible that overexpression of several non-native proteins—among which a macromolecular complex targeted to the inner membrane—could have had destabilizing effects on the expression strain, leading to increased cell lysis and spirosome release [35,36].

### *S*. *pneumoniae* spirosomes share significant structural similarity with other bacterial proteinaceous filaments

**Fig 5:**
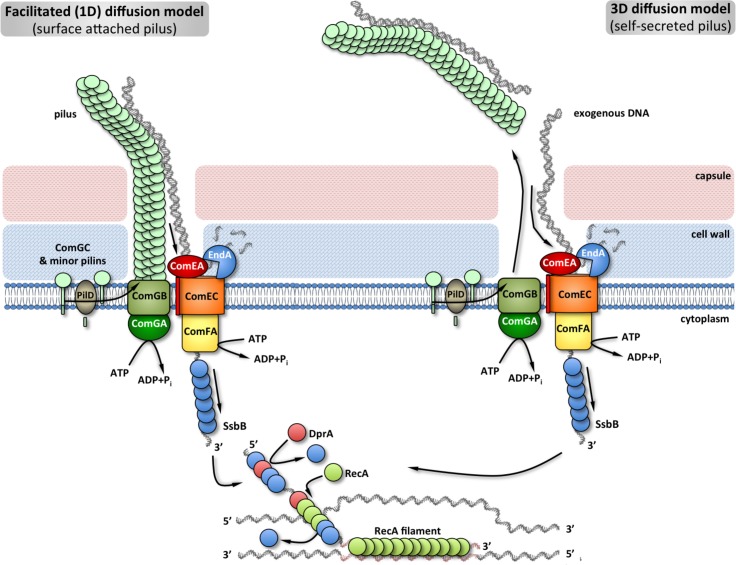
Comprehensive model of pneumococcal natural transformation [5–7,10,61]: upon competence induction a transformation pilus is expressed on the cell surface. It is a ~ 5–6 nanometers wide, several micrometers long helical filament, morphologically and compositionally similar to T4P in Gram-negative bacteria. While its major component is the ComGC pilin, minor pilins ComGD-G as well as the biosynthetic ComGA-ComGB platform are required for pilus biogenesis. Upon expression, pilins are processed by the prepilin peptidase PilD. The mature pilus would serve as an initial ‘trap’ for exogenous DNA or, alternatively, self-secrete. In the former case, transforming DNA would then be guided to the uptake machinery either through pilus retraction, charge-based gliding on the pilus helical lattice (one-dimensional diffusion), or another as yet uncharacterized mechanism. In a hypothetical self-secretion scenario, transforming DNA would have to find the uptake machinery through a three-dimensional diffusion process, while avoiding inhibitory interactions with the secreted pili. Cell-surface binding by the DNA receptor ComEA and single-strand conversion by the nuclease EndA would then guide transforming DNA to the ComEC transporter. The translocase ComFA in partnership with cofactors would power transmembrane transport through ATP hydrolysis. Upon entry in the cytosol the single-stranded DNA would be immediately protected and prepared for recombination.

In our quest to identify the structures observed by Balaban and colleagues [8], we initially hypothesized that they were randomly released RecA nucleofilaments due to their striking similarity to polymerized RecA homologs from other bacterial and eukaryotic species. Such structures have been extensively documented in the literature: forming characteristic helical coils, RecA filaments can be more or less extended depending on the presence and type of DNA and small-molecule ligands [37–39]. Essential but not exclusive to natural transformation, cytosolic RecA is massively expressed during competence and its polymerization on the incoming single-stranded DNA is key to DNA protection and subsequent integration in the genome (Fig 5) [40]. Nevertheless, while *ΔrecA* cells display normal DNA uptake during competence, RecA-based recombination plays pivotal role in DNA repair throughout the bacterial life cycle [40,41]. This indicates that pilus-dependent DNA uptake and RecA polymerization are intrinsically uncoupled and could have explained the continuous release of ‘plaited’ filaments in the pilus-defficient strains. We were indeed able to detect RecA release in the medium of competence-induced cultures of both wild-type and *ΔcomGB* cells (S3 Fig panel *A*). Also, although with slightly different parameters in terms of helical width and pitch, the ‘plaited’ filaments were structurally similar to both the coiled structure of a eukaryotic RecA homolog (S3 Fig panel *B*) [37], as well as to *in vitro* reconstituted RecA^*S*.*pneumoniae*^ nucleofilaments (S3 Fig panel *C*). Nevertheless, release of the characteristic polymers persisted in a *ΔrecA* strain (S3 Fig panel *D*) and we were unable to detect the protein in the filament-enriched fractions following purification (S1 Table).

It is therefore important to note that macromolecular organization in helical filaments is not uncommon among proteins from both the bacterial and other taxa. These include but are not limited to nucleic acid-binding proteins, cytoskeletal elements, building blocks of cell surface appendages and phage capsid subunits [39,42–44]. This, together with the markedly different helical parameters of the highly conserved *E*. *coli* spirosomes shown here underscores the fact that limited-view, low-resolution morphology imaging and bulk biochemical experiments alone are often insufficient to deduce the nature of macromolecular assemblies. Rather, a combination of orthogonal approaches that spans the different resolution levels and integrates genetic, biochemical and structural data in a meaningful way is generally warranted to avoid false-positive or otherwise erroneous results.

### *S*. *pneumoniae* requires a long, T4P-like transformation pilus for DNA uptake

Taken together, our data rule out the existence of short ‘plaited’ transformation pili in competent pneumococci and reassert the expression of a long, 5–6 nanometer wide appendage, structurally and compositionally similar to T4P in Gram-negative bacteria [10]. This finding bridges Gram-negative and Gram-positives DNA uptake systems and provides a comprehensive picture of this major lateral gene transfer event. Indeed, a recent study showed the existence of a T4-pilus on competent *V*. *cholerae*, which shares many features with the pneumococcal transformation pilus: competence-induced expression, prerequisite for DNA uptake, and roughly a single copy per cell [45].

Apart from morphology alone, however, it is interesting to discuss the probable mechanism through which the transformation pili secure DNA entry into competent pneumococci. Although expression of any type of pilus would require overcoming the physical barriers of cell-wall peptidoglycan and overlaying capsule—and thus possibly facilitate DNA entry—the similarities among transformation pili of Gram-positive and Gram-negative bacteria suggest that naturally transformable species might have evolved a conserved and more sophisticated mechanism of pilus function than simple cell-wall destabilization.

In agreement with a long-standing ‘pseudo-pilus’ hypothesis, Balaban and colleagues proposed a model in which the transformation pili self-secrete in the medium of competent *S*. *pneumoniae*, thus opening gateways in the cell wall peptidoglycan for passive exogenous DNA entry [8,9]. Their hypothesis was supported by the observation that ComGC found in the supernatant of different *S*. *pneumoniae* strains after centrifugation correlates with the peak of transformation efficiency [8]. Since we previously showed that transformation pilus expression is absolutely required for DNA uptake, it is not surprising to observe correlation between extracellular ComGC and transformation efficiency [10]. However, ComGC release in culture supernatants can be a result from both cell lysis and/or compromised pilus integrity. As we showed previously, pneumococcal transformation pili are fairly sensitive to mechanical stress and short vortexing and centrifugation are routinely used for their shearing and isolation [10,12]. Such mechanical forces, however, are unlikely to be exerted in nature, where competent pneumococci are typically cushioned in protective biofilm matrix [46].

As we have conducted only single time point visualization experiments, it is theoretically possible that the expresses transformation pili eventually detach from the cell to open entry pores for transforming DNA (Fig 5). However, the sheer size and ATP-dependent assembly of the transformation pilus makes such self-secretion hypothesis unlikely: the observed long native pili would be energetically taxing on the cells if their sole function were to be ejected prior to DNA uptake. Finally, it has been previously reported that native transformation pili bind and co-purify with DNA already present in the cell culture and that DNA binding at the surface of competent pneumococci is abolished in a pilus-deficient strain [10,47]. DNA-binding is also conserved in the homologous T4P of Gram-negative bacteria [14,48,49]. In such a DNA-binding context, pilus release would actually inhibit transformation by titrating out DNA available for uptake (Fig 5). This once again argues against a self-secreting mechanism of function and reinforces a cell-surface attached role for the pilus in transformation.

While no mechanistic or quantitative data on DNA binding by the pilus are available, electron microscopy showed extensive contact interfaces between the long transformation pili and DNA chains [10]. It is therefore plausible that multiple weak interactions along the helical pilus lattice stabilize this interaction and allow its reversal upon DNA uptake. Such a scenario would also explain why no DNA binding to a non-polymerizing ComGC truncation has ever been detected [8,50]. Even more interesting, however, is the question of how pilus-bound DNA gets brought to the DNA uptake machinery in the cell membrane. In Gram-negative bacteria, T4P-bound DNA is proposed to be actively hauled to the cell by rapid bottom-up pilus depolymerization powered by a dedicated retraction ATPase [14]. Although a similar mechanism has been proposed for *S*. *pneumoniae* and other transformable Gram-positive bacteria, pneumococci lack homologous retraction ATPase and are likely to utilize a distinct mechanism for DNA entry. In addition, transforming DNA uptake occurs at much lower speeds in Gram-positive bacteria than Gram-negative T4P retraction [51,52].

Many sequence-specific DNA binding proteins can scan DNA for their target sites at speeds several orders of magnitude higher than the upper limit for a three-dimensional diffusion-controlled process [53]. This can generally be achieved by at least two passive mechanisms, which involve sequence non-specific DNA binding and subsequent translocation of the protein along the DNA: 1) charge-based protein sliding, where the protein engages in a one-dimensional random walk along the DNA in search of its target, and 2) direct intersegment transfer, where the protein can bind and hop between two remote regions on the DNA without losing the non-specifically bound state [53]. Although we can not exclude the involvement of an unidentified retraction ATPase or additional receptor proteins in exogenous DNA uptake, we favor a model where the pneumococcal transformation pilus provides a similar facilitated diffusion framework (Fig 5). By preserving multiple dynamic non-specific interactions with the pilus, transforming DNA would overcome the thermodynamic limitations of a three-dimensional diffusion process until it passively finds the membrane associated uptake machinery and becomes actively pumped in the cell (Fig 5).

## Materials and Methods

### Spirosome release visualization by electron microscopy (EM)

*Streptococcus pneumoniae* spirosomes were observed in both competent and non-competent cells. For competence induction cells were grown in microaerobic conditions, without agitation, at 37°C in Casamino Acid-Tryptone (CAT) medium supplemented with 0.2% glucose, 15mM dipotassium phosphate, 3mM sodium hydroxide and 1mM calcium chloride and adjusted to pH 7.8. Competence was triggered by the addition of Competence Stimulating Peptide (CSP) at OD_600_ = 0.15 for 10–30 min. Non-competent pneumococci and *Escherichia coli* cells were grown similarly in LB to OD_600_ = 0.3 and OD_600_ = 0.6, respectively. *Clostridium difficile* cells were grown at 37°C under strict anaerobic conditions on Tryptone-Yeast extract-Glucose (TYG) plates supplemented with 0.1% thioglycolate. *Streptococcus sanguinis* cells were grown anaerobically, without agitation, at 37°C in CAT medium to OD_600_ = 0.3. For spirosome visualization cells were scraped off the plates or pelleted by centrifugation and resuspended in TBS (50 mM Tris-HCl pH 7.6, 150 mM NaCl) at ~ 5 μl TBS per milliliter of culture at OD_600_ = 0.3. 5 μl drops of each suspension were then placed directly on glow discharged carbon coated grids (EMS, USA) for 1 minute. The grids were then blot-dried on filter paper, washed on a drop of ultrapure water, and negatively stained with 2% uranyl acetate in water. Specimens were examined on an FEI Tecnai T12 BioTWIN LaB6 electron microscope operating at 120 kV at nominal magnifications of 18500–68000 and 1–3 μm defocus. Images were recorded on a Gatan Ultrascan 4000 CCD camera.

### Transformation efficiency assay

An *adhE* deletion (strain AD001) was introduced in the R1501 genetic background by transformation with a DNA cassette carrying a kanamycin resistance gene inserted between two ~1000 base pair fragments corresponding to the *S*. *pneumoniae* genomic regions flanking *adhE*. Briefly, the genomic region upstream from the AdhE open reading frame was amplified using forward and reverse primers 5’-ACA TGG CAA TCC GAT TCA TAA GGG G-3’ and 5’-GCC ATC TAT GTG TCG GAA CGA TAT CCT TTG TTA ATT TTT TCA CAA GTT TAT TAT AAC G-3’, respectively, while the genomic region downstream of the *adhE* gene was amplified the following primer pair 5’-AAA ATG TGT TTT TCT TTG TTT TGT TTA TCA GTC TAG AAG CAA GAC AAA AAC TCA A-3’ and 5’-TTG CTA TTT ATG CAT GCA GAA GAC CAA ATG-3’. A third pcr reaction was used to amplify a kanamycin-resistance gene using the pR411 plasmid as template DNA [54] and forward and reverse primers 5’-AGG ATA TCG TTC CGA CAC ATA GAT GGC GTC GCT AGT-3’ and 5’-GCT TCT AGA CTG ATA AAC AAA ACA AAG AAA AAC ACA TTT TTT TGT CAA AAT TCG TTT-3’, carrying complementarity to the 3’-end of the *adhE*-upstream and 5’-end of the *adhE*-downstream fragments, respectively. The three pcr products were then assembled using overlap extension PCR and the purified DNA cassette was used for transformation of competence-induced *S*. *pneumoniae* R1501 cells. *adhE*-null mutants (strain AD001) were positively selected by growth in the presence of kanamycin (60 μg/ml) and *adhE* deletion was confirmed independently by DNA sequencing and western blot detection using an anti-AdhE antibody. For all transformation experiments, competence was triggered as above at OD_600_ = 0.15 for 10 minutes, followed by DNA addition and 20 minute incubation at 30°C. Transformants were selected on Columbia Agar supplemented with 5% horse blood and appropriate antibiotics. For the transformation efficiency assays, cells were transformed with 100 ng of a DNA cassette, amplified from *S*. *pneumoniae* R304 genomic DNA and containing the streptomycin resistance gene *str41*. Bacteria were plated in the presence and absence of streptomycin (100 μg/ml) and incubated at 37°C overnight before colony counting.

### Spirosome enrichment and purification

All steps of the purification protocol were performed at 4°C. 8L of *S*. *pneumoniae* culture grown in LB to OD_600_ = 0.3 or 4L of *E*. *coli* culture grown to OD_600_ = 0.6 were pelleted by centrifugation (20 min at 5000 g) and resuspended in 6 ml of cold TBS, vortexed briefly and centrifuged to remove the bulk of intact cells and debris (10 min at 12000 g followed by 15 min at 50000 g). Triton x-100 was added to the supernatant at final concentration of 0.25%. Following 30 minute agitation for solubilization of remaining membrane fragments, the samples were filtered through a 0.45 μm cellulose acetate filter (Corning) and centrifuged for 1h at 125000 g for spirosome pelleting. After careful removal of the supernatant, the pellet was resuspended in 50 μl TBS, re-filtered and loaded on a Superose 6 3.2/300 size exclusion column (GE Healthcare). Spirosome enriched fractions were found to elute with the void volume. Sample preparation for electron microscopy was performed as above.

### Proteomics analysis

Trypsin digestion was performed as described previously [55] and the digests were analyzed under standard conditions on an LTQ-Orbitrap Velos (Thermo Fisher, Bremen) equipped with Ultimate 3000 nano-HPLC (Dionex). Briefly, tryptic peptides were desalted and separated on a C-18 nano-HPLC column under a gradient of acetonitrile. Minimum signal threshold for triggering an MS/MS event was set to 5000 counts. After a survey scan, the 10 most intense precursor ions were selected for CID fragmentation (top10). Raw files were processed with MaxQuant 1.4.1.2 [56]. Protein identification was done using Andromeda against a *Streptococcus pneumoniae* (strain ATCC BAA-255 / R6) (Taxonomy 171101–1947 proteins) or *Escherichia coli* (strain K12 / MC4100 / BW2952) (Taxonomy 595496–4043 proteins) database. A false-discovery rate of 1% was used for both peptide and protein identification. Reverse and contaminant proteins were excluded and only proteins identified with a minimum of 2 peptides were considered.

### Single-particle EM data processing

Spirosomes and transformation pili were visualized by EM as above at nominal magnifications of 49000 and 68000, respectively. The contrast transfer function parameters were assessed using CTFFIND3 [57], and the phase flipping was done in SPIDER [58]. Linear filament segments were boxed with e2helixboxer and single particle stacks were generated for each dataset (EMAN2 [59]): 4309 particles for the transformation pilus (134x134 pixel box, 1.5 Å/pixel), 728 particles for the *S*. *pneumoniae* spirosomes (180x180 pixel box, 2.2 Å/pixel) and 555 particles for the *E*. *coli* spirosomes (180x180 pixel box, 2.2 Å/pixel). Normalization, centering, multi-reference alignment, multi-statistical analysis, and classification (15–40 particles per class) were done in IMAGIC-4D (Image Science Software, GmbH). IMAGIC-4D was also used for generation of two-dimensional reprojections of the T2SS pilus and the *G*. *thermoglucosidasius* spirosome.

### Heterologous expression and purification of AdhE^*S*.*pneumoniae*^

The coding sequence for AdhE^*S*.*pneumoniae*^ was cloned in-frame in a pET21a vector for heterologous expression of C-terminally hexahistidine-tagged protein in *E*. *coli*. Briefly, the AdhE^*S*.*pneumoniae*^ open reading frame was PCR-amplified from *S*. *pneumoniae* R1501 genomic DNA using forward and reverse primers 5’-CAT ATG AAA GCT ATG GAG GAA AAT ATG GCT G-3’ and 5’-GCG GCC GCT TTA CGG CGT CCT GGT CTT TCT TTG-3’, respectively. The pET21a vector was pcr-amplified using forward and reverse primers 5’-GGA CGC CGT AAA GCG GCC GCA CTC GAG CAC CAC CAC-3’ and 5’-CAT ATT TTC CTC CAT AGC TTT CAT ATG TAT ATC TCC TTC TTA-3’, respectively. 90 ng of linearized vector DNA were incubated in 1:1 molar ratio with the AdhE pcr product in an In-Fusion cloning reaction (Clontech) and transformed into Top10 cells (Invitrogen). Plasmid DNA was purified from individual clones, verified for AdhE coding region insertion and used for transformation in BL21 (DE3) cells (Invitrogen). For expression, transformed BL21 (DE3) cells were grown under agitation at 37°C in LB to OD_600_ = 0.6 and expression was induced with 0.7 mM IPTG for 2h. Cells were pelleted by centrifugation (4500 g for 20 min), resuspended in TBS supplemented with c*O*mplete Protease Inhibitor Cocktail (Roche) and disrupted by sonication. Cellular debris were removed by centrifugation (20 000 g for 30 min) and the lysates were incubated with batch Talon metal affinity resin (Clontech) for 30 min at room temperature. The resin was extensively washed (TBS, 15 mM imidazole) and bound protein was eluted with TBS supplemented with 140 mM Imidazole. For EM observation eluted protein was immediately applied on glow-discharged continuous carbon grids, stained, and imaged as above.

### Immunopurification of spirosomes

For AdhE spirosome pull-down, a strain carrying an additional competence-inducible FLAG-tagged ectopic copy of the *adhE* gene was constructed. Briefly, the *adhE* gene was PCR-amplified using *S*. *pneumoniae* R1501 genomic DNA as template and primer pair 5’-GAG GAA GAA ACC ATG TTG AAA GCT ATG GAG GAA AAT ATG GCT GAT AAA AAA AC-3’ and 5’-AAA ATC AAA CGG ATC TTA CTT GTC ATC GTC ATC CTT GTA ATC TTT ACG GCG TCC TGG TCT TTC TTT G-3’, the latter designed to add a C-terminal FLAG tag to the encoded protein. In parallel, pCEPx vector DNA [60] was digested with NcoI and BamHI restriction enzymes. 90ng of the linearized vector were incubated in 1:1 molar ratio with the *adhE-FLAG* pcr product in an In-Fusion cloning reaction (Clontech) and transformed into Top10 *E*. *coli* cells (Invitrogen). Plasmid DNA was purified from individual clones, verified for AdhE-FLAG coding region insertion, and transformed into competence-induced R1501 cells, followed by selection with kanamycin (Kan). The resulting SO007 strain was sequence-verified for the pCEPx-derived *adhE-FLAG*–*KanR* cassette recombination [60] and cultured in CAT medium to OD_600_ = 0.15. CSP-induced and non-induced cells were pelleted by centrifugation and resuspended in TBS by brief vortexing in the presence of millimeter-sized glass beads for increased cell lysis. Cells were pelleted again, and the supernatants were incubated with anti-FLAG M2 affinity resin (Sigma-Aldrich A2220) for 1h at RT and under agitation. After washing with TBS, the resin-bound fraction was eluted by the addition of 100 μg/mL 3X FLAG peptide (Sigma Aldrich F4799) and mixing for additional 15 min at 4°C. The samples were then subjected to SDS-PAGE and EM analyses.

### In vitro reconstitution of RecA filaments

2.5 μM purified RecA^*S*. *pneumoniae*^ was incubated with 50 mM KCl, 0.5 mM DTT, 10 mM Tris-HCl pH 7.5, 2 mM magnesium acetate, and 2 mM ATP-γ-S for 15 minutes at 37°C. RecA nucleofilament formation was induced by the addition of a 54-nucleotide-long single-stranded DNA fragment or single-stranded M13mp18 DNA (New England Biolabs) at nucleotide:RecA monomer ratio of 3:1. After additional 15 minutes at 37°C, the samples were prepared for EM observation as above.

## Supporting Information

S1 Fig Spirosome formation by the ComGC^E20V^ mutant. (TIF)

S2 Fig Color-coded surface-residue conservation across sequences from Table 1 mapped on the *G*. *thermoglucosidasius* AdhE monomer and visualized in UCSF Chimera [19,28]. (TIF)

S3 Fig Structural similarity between spirosomes and RecA nucleofilaments. **(A)** Western blot detection of RecA release in the medium of competence-induced *S*. *pneumoniae* cells. **(B)** Representative class average of *S*. *pneumoniae* spirosomes from culture supernatant compared to the reprojected nucleofilament structure of RecA homolog hDmc1 [37]. **(C)** *In vitro* reconstituted RecA^*S*. *pneumoniae*^—ssDNA nucleofilament (left) compared to a spirosome from culture supernatant (right). Scale bars 10 nm. **(D)** Unabolished spirosome release in *ΔrecA S*. *pneumoniae* culture.
(TIF)

S1 Table Bacterial strains. (DOCX)

S2 Table Mass spectrometry analysis of the purified *S*. *pneumoniae* spirosome fraction. (DOCX)

S3 Table Mass spectrometry analysis of the purified *E*. *coli* spirosome fraction. (DOCX)
