# Supplementary figures and images for: Conserved Streptococcus pneumoniae Spirosomes Suggest a Single Type of Transformation Pilus in Competence
Source: PLoS Pathog. 2015 Apr 15;11(4):e1004835. doi: 10.1371/journal.ppat.1004835 (PMC4398557; doi:10.1371/journal.ppat.1004835)

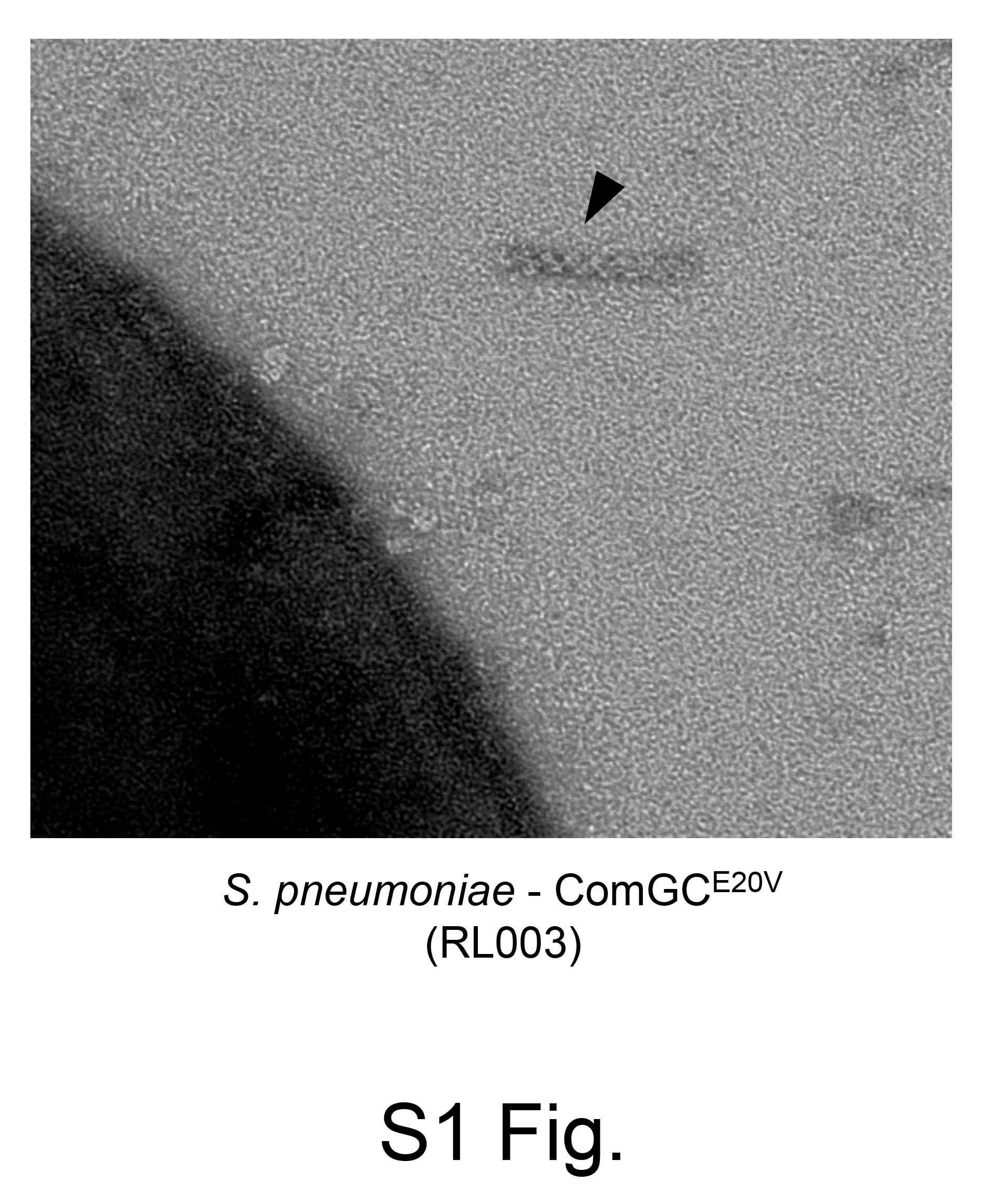

Supplement: S1 Fig — (TIF) [file ppat.1004835.s001.tif]

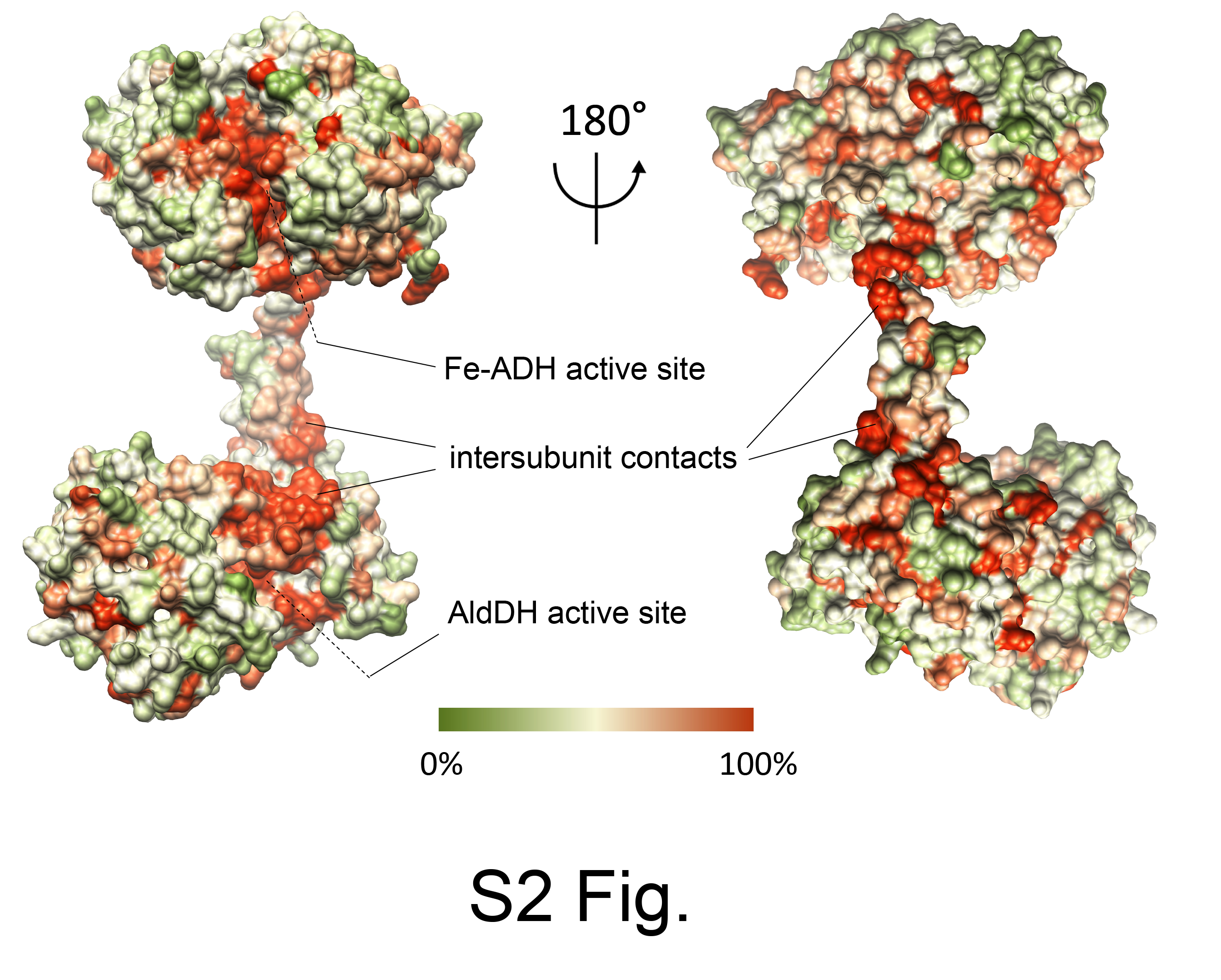

Supplement: S2 Fig — (TIF) [file ppat.1004835.s002.tif]

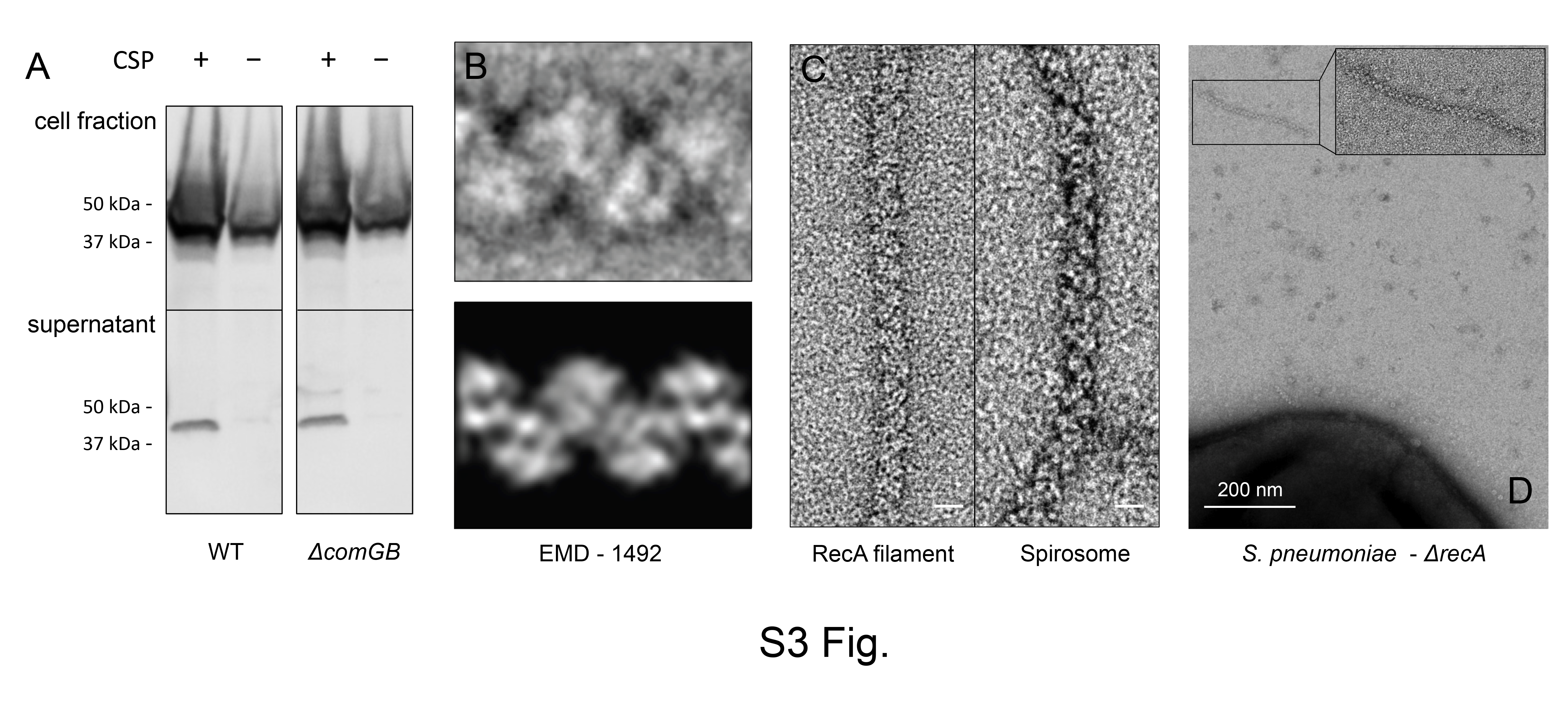

Supplement: S3 Fig — (A) Western blot detection of RecA release in the medium of competence-induced S. pneumoniae cells. (B) Representative class average of S. pneumoniae spirosomes from culture supernatant compared to the reprojected nucleofilament structure of RecA homolog hDmc1 [37]. (C) In vitro reconstituted RecAS. pneumoniae—ssDNA nucleofilament (left) compared to a spirosome from culture supernatant (right). Scale bars 10 nm. (D) Unabolished spirosome release in ΔrecA S. pneumoniae culture. (TIF) [file ppat.1004835.s003.tif]
