# Supplementary material for: Conserved Streptococcus pneumoniae Spirosomes Suggest a Single Type of Transformation Pilus in Competence
Source: PLoS Pathog. 2015 Apr 15;11(4):e1004835. doi: 10.1371/journal.ppat.1004835 (PMC4398557; doi:10.1371/journal.ppat.1004835)
Supplement: S1 Table — (DOCX) [file ppat.1004835.s004.docx]

| **S1 Table**: Bacterial strains | | | |
| --- | --- | --- | --- |
| **Organism** | **Strain** | **Genotype / Relevant feature** | **Reference** |
| ***S. pneumoniae*** | R1501 | R6 derivative but *∆comC* | [1] |
|  | R2456 | R1501 but CEP_x_*-GFP, recA::ermAM; Kan^R^* | Claverys strain collection |
|  | R1916 | R1501 but *ssbB::luc (ssbB^+^), comGA::kan; Cm^R^, Kan^R^* | Claverys strain collection |
|  | R1918 | R1501 but *comGB::kan; Kan^R^* | [2] |
|  | RL001 | R1501 but CEP_x_-*comGC-FLAG; Kan^R^* | [3] |
|  | RL003 | R1501 but *comGC^E20V^* | [3] |
|  | AD001 | R1501 but *∆adhE* (*adhE::kan*) | This study |
|  | SO007 | R1501 but CEP_x_-*adhE-FLAG* | This study |
| ***S. sanguinis*** | SK36 | wild-type | [4] |
|  | Ssx_0068 | SK36 but *∆adhE* (*ssa_0068::aphA-3; Kan^R^*) | [4] |
| ***E. coli*** | BL21(DE3) | *F- ompT hsdSB (rB-mB-) gal dcm* (DE3) | Invitrogen™ |
|  | Top10 | *F- mcrA Δ(mrr-hsdRMS-mcrBC) φ80lacZΔM15 ΔlacX74 recA1 araD139 Δ(ara-leu)7697 galU galK rpsL (StrR) endA1 nupG* | Invitrogen™ |
| ***C. difficile*** | 630 Delta erm | 630 derivative | [5] |

1. Dagkessamanskaia A, Moscoso M, Henard V, Guiral S, Overweg K, et al. (2004) Interconnection of competence, stress and CiaR regulons in Streptococcus pneumoniae: competence triggers stationary phase autolysis of ciaR mutant cells. Molecular microbiology 51: 1071-1086.

2. Havarstein LS, Martin B, Johnsborg O, Granadel C, Claverys JP (2006) New insights into the pneumococcal fratricide: relationship to clumping and identification of a novel immunity factor. Molecular microbiology 59: 1297-1307.

3. Laurenceau R, Pehau-Arnaudet G, Baconnais S, Gault J, Malosse C, et al. (2013) A type IV pilus mediates DNA binding during natural transformation in Streptococcus pneumoniae. PLoS pathogens 9: e1003473.

4. Xu P, Ge X, Chen L, Wang X, Dou Y, et al. (2011) Genome-wide essential gene identification in Streptococcus sanguinis. Scientific reports 1: 125.

5. Sebaihia M, Wren BW, Mullany P, Fairweather NF, Minton N, et al. (2006) The multidrug-resistant human pathogen Clostridium difficile has a highly mobile, mosaic genome. Nature genetics 38: 779-786.
