# Supplementary material for: Conserved Streptococcus pneumoniae Spirosomes Suggest a Single Type of Transformation Pilus in Competence
Source: PLoS Pathog. 2015 Apr 15;11(4):e1004835. doi: 10.1371/journal.ppat.1004835 (PMC4398557; doi:10.1371/journal.ppat.1004835)
Supplement: S2 Table — (DOCX) [file ppat.1004835.s005.docx]

| **S2 Table**: Purified *S. pneumoniae* spirosome fraction | | | | | |
| --- | --- | --- | --- | --- | --- |
| **Accession** | **Protein** | **Unique peptides** | **Sequence coverage** | **Mw** | **PEP** |
| Q8DNA5 | AdhE | 35 | 47.4 % * | 98.2 kDa | 0 |
| P0A4D8 | Exp9 | 12 | 32.1 % | 58.8 kDa | 4.77E-120 |
| Q8CWR9 | RpsA | 12 | 33.8 % | 43.9 kDa | 2.88E-71 |
| P64031 | Tuf | 10 | 27.1 % | 44.0 kDa | 1.58E-43 |
| P66393 | RpsM | 7 | 37.2 % | 13.4 kDa | 1.07E-41 |
| P66566 | RpsD | 7 | 31 % | 23.0 kDa | 5.33E-33 |
| Q8CWV5 | RplB | 7 | 37.9 % | 29.9 kDa | 9.29E-77 |
| P66096 | RplA | 5 | 21.4 % | 24.5 kDa | 8.07E-43 |
| Q8CWV8 | RplC | 5 | 21.6 % | 22.2 kDa | 2.78E-59 |
| Q8DQV2 | InfB | 4 | 4.6 % | 102.9 kDa | 8.66E-13 |
| P66113 | RplT | 3 | 28.6 % | 13.7 kDa | 8.93E-38 |
| P66603 | RpsF | 3 | 42.7 % | 11.2 kDa | 1.42E-44 |
| Q8CWU4 | RpsI | 3 | 17.7 % | 14.2 kDa | 3.04E-16 |
| Q8CWU6 | RpsG | 3 | 29.5 % | 17.8 kDa | 6.97E-48 |
| Q8CWV7 | RplD | 3 | 19.3 % | 22.1 kDa | 6.18E-37 |
| Q8DNJ0 | RluB | 3 | 14.6 % | 27.1 kDa | 1.00E-25 |
| P0A476 | RplP | 2 | 18.2 % | 15.4 kDa | 2.45E-04 |
| P0A4A8 | RpsL | 2 | 14.6 % | 15.1 kDa | 6.18E-13 |
| P0A4C4 | RpsC | 2 | 13.8 % | 24.0 kDa | 1.79E-07 |
| P60630 | RplX | 2 | 29.7 % | 11.0 kDa | 1.84E-14 |
| P66340 | RpsJ | 2 | 24.5 % | 11.6 kDa | 1.87E-09 |
| P66473 | RpsR | 2 | 17.7 % | 9.2 kDa | 1.12E-04 |
| Q8CWV3 | RplF | 2 | 14.6 % | 19.4 kDa | 1.88E-16 |
| Q8DQT0 | Pnp | 2 | 2.8 % | 81.0 kDa | 8.53E-12 |
| Q8DQH1 | RheB | 2 | 5.6 % | 50.8 kDa | 7.77E-04 |
| Q8DQR3 | Spr0538 | 2 | 5.1 % | 61.2 kDa | 2.92E-31 |
| Q8DRH7 | Spr0125 | 2 | 3.9 % | 66.5 kDa | 1.19E-03 |

* AdhE sequence coverage (grey)

MKAMEENMAD KKTVTPEEKK LVAEKHVDEL VQKALVALEE MRKLNQEQVD YIVAKASVAA LDAHGELALH AFEETGRGVF EDKATKNLFA CEHVVNNMRH TKTVGVIEED DVTGLTLIAE PVGVVCGITP TTNPTSTAIF KSLISLKTRN PIVFAFHPSA QESSAHAARI VRDAAIAAGA PENCVQWITQ PSMEATSALM NHEGVATILA TGGNAMVKAA YSCGKPALGV GAGNVPAYVE KSANIRQAAH DIVMSKSFDN GMVCASEQAV IIDKEIYDEF VAEFKSYHTY FVNKKEKALL EEFCFGVKAN SKNCAGAKLN ADIVGKPATW IAEQAGFTVP EGTNILAAEC KEVGENEPLT REKLSPVIAV LKSESREDGI TKARQMVEFN GLGHSAAIHT ADEELTKEFG KAVKAIRVIC NSPSTFGGIG DVYNAFLPSL TLGCGSYGRN SVGDNVSAIN LLNIKKVGRR RNNMQWMKLP SKTYFERDSI QYLQKCRDVE RVMIVTDHAM VELGFLDRII EQLDLRRNKV VYQIFADVEP DPDITTVNRG TEIMRAFKPD TIIALGGGSP MDAAKVMWLF YEQPEVDFRD LVQKFMDIRK RAFKFPLLGK KTKFIAIPTT SGTGSEVTPF AVISDKANNR KYPIADYSLT PTVAIVDPAL VLTVPGFVAA DTGMDVLTHA TEAYVSQMAS DYTDGLALQA IKLVFENLES SVKNADFHSR EKMHNASTIA GMAFANAFLG ISHSMAHKIG AQFHTIHGRT NAILLPYVIR YNGTRPAKTA TWPKYNYYRA DEKYQDIARM LGLPASTPEE GVESYAKAVY ELGERIGIQM NFRDQGIDEK EWKEHSRELA FLAYEDQCSP ANPRLPMVDH MQEIIEDAYY GYKERPGRRK
