# Supplementary material for: Conserved Streptococcus pneumoniae Spirosomes Suggest a Single Type of Transformation Pilus in Competence
Source: PLoS Pathog. 2015 Apr 15;11(4):e1004835. doi: 10.1371/journal.ppat.1004835 (PMC4398557; doi:10.1371/journal.ppat.1004835)
Supplement: S3 Table — (DOCX) [file ppat.1004835.s006.docx]

| **S3 Table**: Purified *E. coli* spirosome fraction | | | | | |
| --- | --- | --- | --- | --- | --- |
| **Accession** | **Protein** | **Unique peptides** | **Sequence coverage** | **Mw** | **PEP** |
| C4ZTT2 | AdhE | 41 | 57.7 % * | 96.1 kDa | 0 |
| C5A0S7 | RpoB | 23 | 22.9 % | 150.6 kDa | 2.43E-261 |
| C5A0S8 | RpoC | 18 | 17 % | 155.2 kDa | 2.62E-214 |
| C4ZQ83 | OmpA | 13 | 44.5 % | 37.2 kDa | 7.07E-163 |
| C4ZRK8 | AceF | 12 | 22.2 % | 66.1 kDa | 1.27E-151 |
| C4ZU58 | OmpC | 13 | 48 % | 40.4 kDa | 1.61E-167 |
| C4ZUF0 | RpoA | 7 | 28 % | 36.5 kDa | 7.42E-49 |
| C4ZRK7 | AceE | 4 | 5.3 % | 99.7 kDa | 6.86E-62 |
| C5A0S0 | TufB | 7 | 23.6 % | 43.3 kDa | 2.26E-65 |
| C5A0R5 | BtuB | 3 | 7 % | 68.4 kDa | 4.88E-46 |
| C4ZRK9 | Lpd | 4 | 13.1 % | 50.7 kDa | 1.78E-85 |
| C4ZV09 | OmpT | 3 | 14.8 % | 35.6 kDa | 3.12E-30 |
| C4ZQ55 | OmpF | 5 | 17.7 % | 39.3 kDa | 3.11E-43 |
| C4ZTF1 | RdgC | 3 | 15.8 % | 34.0 kDa | 3.32E-58 |
| C4ZS88 | YmgG | 4 | 62.3 % | 10.8 kDa | 7.39E-86 |
| C4ZTG8 | Tsx | 3 | 13.6 % | 33.6 kDa | 3.95E-15 |
| C4ZXY6 | OmpX | 4 | 25.7 % | 18.6 kDa | 4.35E-73 |
| C4ZTW7 | TopA | 2 | 3 % | 97.3 kDa | 3.89E-09 |
| C4ZYA4 | SlyB | 3 | 31 % | 15.6 kDa | 1.04E-35 |
| C5A0S6 | RplL | 2 | 19 % | 12.3 kDa | 3.28E-11 |
| C4ZYD9 | Lpp | 2 | 33.3 % | 8.3 kDa | 2.31E-47 |

* AdhE sequence coverage (grey)

MAVTNVAELN ALVERVKKAQ REYASFTQEQ VDKIFRAAAL AAADARIPLA KMAVAESGMG IVEDKVIKNH FASEYIYNAY KDEKTCGVLS EDDTFGTITI AEPIGIICGI VPTTNPTSTA IFKSLISLKT RNAIIFSPHP RAKDATNKAA DIVLQAAIAA GAPKDLIGWI DQPSVELSNA LMHHPDINLI LATGGPGMVK AAYSSGKPAI GVGAGNTPVV IDETADIKRA VASVLMSKTF DNGVICASEQ SVVVVDSVYD AVRERFATHG GYLLQGKELK AVQDVILKNG ALNAAIVGQP AYKIAELAGF SVPENTKILI GEVTVVDESE PFAHEKLSPT LAMYRAKDFE DAVEKAEKLV AMGGIGHTSC LYTDQDNQPA RVSYFGQKMK TARILINTPA SQGGIGDLYN FKLAPSLTLG CGSWGGNSIS ENVGPKHLIN KKTVAKRAEN MLWHKLPKSI YFRRGSLPIA LDEVITDGHK RALIVTDRFL FNNGYADQIT SVLKAAGVET EVFFEVEADP TLSIVRKGAE LANSFKPDVI IALGGGSPMD AAKIMWVMYE HPETHFEELA LRFMDIRKRI YKFPKMGVKA KMIAVTTTSG TGSEVTPFAV VTDDATGQKY PLADYALTPD MAIVDANLVM DMPKSLCAFG GLDAVTHAME AYVSVLASEF SDGQALQALK LLKEYLPASY HEGSKNPVAR ERVHSAATIA GIAFANAFLG VCHSMAHKLG SQFHIPHGLA NALLICNVIR YNANDNPTKQ TAFSQYDRPQ ARRRYAEIAD HLGLSAPGDR TAAKIEKLLA WLETLKAELG IPKSIREAGV QEADFLANVD KLSEDAFDDQ CTGANPRYPL ISELKQILLD TYYGRDYVEG ETAAKKEAAP AKAEKKAKKS A
